# Homopiperazine Derivatives as a Novel Class of Proteasome Inhibitors with a Unique Mode of Proteasome Binding

**DOI:** 10.1371/journal.pone.0060649

**Published:** 2013-04-11

**Authors:** Jiro Kikuchi, Naoya Shibayama, Satoshi Yamada, Taeko Wada, Masaharu Nobuyoshi, Tohru Izumi, Miyuki Akutsu, Yasuhiko Kano, Kanako Sugiyama, Mio Ohki, Sam-Yong Park, Yusuke Furukawa

**Affiliations:** 1 Division of Stem Cell Regulation, Center for Molecular Medicine, Jichi Medical University, Shimotsuke, Tochigi, Japan; 2 Division of Biophysics, Department of Physiology, Jichi Medical University, Shimotsuke, Tochigi, Japan; 3 Division of Hematology, Tochigi Cancer Center, Utsunomiya, Tochigi, Japan; 4 Protein Design Laboratory, Yokohama City University, Yokohama, Kanagawa, Japan; University of Bologna & Italian Institute of Technology, Italy

## Abstract

The proteasome is a proteolytic machinery that executes the degradation of polyubiquitinated proteins to maintain cellular homeostasis. Proteasome inhibition is a unique and effective way to kill cancer cells because they are sensitive to proteotoxic stress. Indeed, the proteasome inhibitor bortezomib is now indispensable for the treatment of multiple myeloma and other intractable malignancies, but is associated with patient inconvenience due to intravenous injection and emerging drug resistance. To resolve these problems, we attempted to develop orally bioavailable proteasome inhibitors with distinct mechanisms of action and identified homopiperazine derivatives (HPDs) as promising candidates. Biochemical and crystallographic studies revealed that some HPDs inhibit all three catalytic subunits (ß 1, ß 2 and ß 5) of the proteasome by direct binding, whereas bortezomib and other proteasome inhibitors mainly act on the ß5 subunit. Proteasome-inhibitory HPDs exhibited cytotoxic effects on cell lines from various hematological malignancies including myeloma. Furthermore, K-7174, one of the HPDs, was able to inhibit the growth of bortezomib-resistant myeloma cells carrying a ß5-subunit mutation. Finally, K-7174 had additive effects with bortezomib on proteasome inhibition and apoptosis induction in myeloma cells. Taken together, HPDs could be a new class of proteasome inhibitors, which compensate for the weak points of conventional ones and overcome the resistance to bortezomib.

## Introduction

The paradigm of cancer treatment has been dramatically changed by the introduction of small molecular compounds that target the “Achilles' heel” of cancer cells [1]. The proteasome is a proteolytic machinery that executes the degradation of polyubiquitinated proteins to maintain cellular homeostasis [2]. Cancer cells are very sensitive to proteotoxic stress because of intracellular protein overload due to rapid cell cycling and apoptosis inhibition. This feature makes proteasome inhibition a unique and effective way to kill cancer cells that can tolerate conventional therapies [3].

Bortezomib is the first proteasome inhibitor (PI) approved for clinical application, which preferentially targets ß1 and ß5 subunits of the proteasome [3], [4]. This drug is particularly effective for multiple myeloma (MM), because it accelerates the unfolded protein response (UPR) via down-regulation of histone deacetylases (HDACs) [5], [6] and targets cell adhesion-mediated drug resistance via down-regulation of very late antigen-4 [7], [8]. Accordingly, bortezomib is now indispensable for the treatment of MM in combination with other anti-cancer drugs including alkylating agents, corticosteroids and HDAC inhibitors [9]–[11].

Although bortezomib therapy is a major advance in clinical oncology, there are at least three major problems to be resolved as soon as possible. First, bortezomib has several possible off-target toxicities [12], [13]. Second, the development of intrinsic and acquired resistance to bortezomib is an emerging problem [14]–[19]. Third, bortezomib should be administered intravenously on biweekly schedules with treatment periods extending for 6 months or more. The development of orally bioavailable PIs with distinct mode of action is a possible way to circumvent these issues.

Homopiperazine-derived compounds have been developed as orally active agents because of their superb bioavailability. Among them, dilazep, an inhibitor of nucleoside transporters, has been clinically used for the treatment of cardiac dysfunction via post-oral administration [20]. Some homopiperazine derivatives (HPDs), such as K-7174 and K-11706, were shown in pre-clinical studies to inhibit cell adhesion [21] and to rescue anemia of chronic disorders via the activation of erythropoietin production *in vitro* and *in vivo* [22], [23]. In addition, K-7174 was reported to exert anti-inflammatory action via induction of the UPR [24]. These observations prompted us to consider that HPDs could be orally active PIs; however, this possibility has not been tested so far. In this study, we demonstrated that HPDs, including K-7174, have the ability to inhibit proteasome activity via different mechanisms of action from bortezomib and other conventional PIs.

## Materials and Methods

### Cells and Cell Culture

We used cell lines from acute lymphoblastic leukemia (KOPM30 and Jurkat), mantle cell lymphoma (HBL-2, Granta519 and NCEB-1), Burkitt lymphoma (Daudi and Namalwa), multiple myeloma (KMS12-BM, RPMI8226, U266, KMM1 and Delta47) [25], acute myeloid leukemia (HL60, KG1a and U937) and chronic myeloid leukemia (K562) in this study. These cell lines were purchased from the Health Science Research Resources Bank (Osaka, Japan) except for KOPM30, which was provided by Dr. Takeshi Inukai (University of Yamanashi, Yamanashi, Japan) [26], and Granta519 and NCEB-1, which were provided by Professor Martin J. S. Dyer (Leicester University, Leicester, UK) [27], and maintained in RPMI1640 medium (Sigma Co., St. Louis, MO) supplemented with 10% heat-inactivated fetal calf serum (Sigma) and antibiotics.

### Drugs

HPDs and bortezomib were provided by Kowa (Tokyo, Japan) and Millennium Pharmaceuticals (Cambridge, MA), respectively. The compounds were dissolved in dimethyl sulfoxide (DMSO), diluted with 0.9% NaCl at appropriate concentrations and stored at −80°C until use. The chemical structures of K-7174 (N,N’-bis-(E)-(5-(3,4,5-trimethoxy-phenyl)-4-pentenyl) homopiperazine) and other HPDs are shown in Table 1.

**Table 1:** Chemical structures of homopiperazine derivatives used in this study.

Compound	Chemical formula	Number of carbon atom chains
K-7174	N,N’-bis-(E)-[5-(3,4,5-trimethoxy-phenyl)-4-pentenyl] homopiperazine	C = 5
K-7259	N,N’-bis-[4-(3,4,5-trimethoxy-phenyl)-butyl] homopiperazine	C = 4
K-7220	N,N’-bis-[3-(3,4,5-trimethoxy-phenyl)-propyl] homopiperazine	C = 3
K-10310	N,N’-bis-(E)-[6-(3,4,5-trimethoxy-phenyl)-4- oxo-5-hexenyl] homopiperazine	C = 6
K-10252	N,N’-bis-(E)-[7-(3,4,5-trimethy-phenyl)-5-oxo-6- heptenyl] homopiperazine	C = 7
K-10228	N,N’-bis-(E)-[5-(3,4,5-trimethyl-phenyl)-4- pentenyl] homopiperazine	C = 5
K-10256	N,N’-bis-(E)-[5-(2,3,4,5-tetramethoxy-phenyl)-4- pentenyl] homopiperazine	C = 5
K-10487	N,N’-bis-(E)-[4-(3,4,5-trimethoxy-naphthyl)-butyl] homopiperazine	C = 5
K-10552	N,N’-bis-(E)-[5-(3,4,5-trimethoxy-phenyl)-4- pentenyl]-6-methoxy-homopiperazine	C = 5

### Cell Proliferation Assay

Cell proliferation was measured by the 3-(4,5-dimethylthiazol-2-yl)-2,5-diphenyltetrazolium (MTT) reduction assay using a Cell Counting Kit (Wako Biochemicals, Osaka, Japan). Absorbance at 450 nm was analyzed with a microplate reader and expressed as a percentage of the value of corresponding untreated cells.

### Immunoblotting

Immunoblotting was carried out according to the standard method using the following antibodies: anti-ubiquitin, anti-ubiquityl histone H2A (Lys119), anti-K48-linked polyubiquitin (Cell Signaling Technology, Beverly, MA), anti-proteasome ß5 subunit (PSMB5) (Enzo Life Sciences, Farmingdale, NY), and anti-GAPDH (Santa Cruz Biotechnology, Santa Cruz, CA) [28].

### 20 S Proteasome Activity Assay

Proteasome activity assays were performed using 20 S proteasome assay kits (Enzo Life Sciences and Cayman Chemical, Ann Arbor, MI). Proteasomal chymotrypsin-like, trypsin-like and caspase-like activities were determined using fluorogenic substrates suc-LLVY-amc, boc-LRR-amc, and z-LLE-amc (Enzo Life Sciences), respectively, with purified human erythrocyte-derived 20 S proteasome (Enzo Life Sciences) or MM cell lysates. Reactions were initiated by enzyme or lysate addition, and monitored for amc product formation at 30°C on a spectrofluorometer (SpectraMax Gemini EM; Molecular Devices, Sunnyvale, CA) using excitation of 360 nm and emission of 480 nm [29], [30].

### X-ray Crystallographic Analysis of the K-7174/proteasome Complex

Single crystals of 20 S proteasome from *Saccharomyces cerevisiae* (Enzo Life Sciences) in complex with K-7174 were grown using the sitting drop vapor diffusion method at 20°C by mixing 8 µl of protein and 8 µl of reservoir solution. The protein concentration used for crystallization was 10 mg/ml in 10 mM Tris-HCl (pH 7.5) and 1 mM EDTA. The reservoir solution contained 4.5% (v/v) 2-methyl-2,4-pentanediol (MPD), 36 mM magnesium acetate, 90 mM morpholino-ethane-sulphonic acid (MES, pH 7.2), 10% (v/v) DMSO, and 12.5 mM K-7174. Crystals were soaked in cryoprotectant buffer containing 30% (v/v) MPD and flash frozen in liquid nitrogen. X-ray data were collected at beamline BL44XU of Spring-8 (Hyogo, Japan) equipped with an MAR CCD detector 225 mm at 100 K under a nitrogen gas stream. The wavelength of the incident X-ray was 1.0 Å. Diffraction data sets were processed with HKL2000, and scaled with SCALEPACK [31]. The crystals belonged to the space group *P2*_1_ with unit cell parameters of *a = *134.26, *b = *301.36, *c = *143.96 and *ß* = 112.9°. The structures were solved by molecular replacement using MOLREP [32] with the previously reported structure of 20 S proteasome (Protein Data Bank code 1RYP) as a starting model. The best solution using data from 40 to 3.5 Å resolution range yielded a correlation coefficient of 0.7 and an *R*-factor of 0.33 for structure, after rigid body refinement. At this stage, crystallographic refinement was pursued in PHENIX [33]. After an initial round of simulated annealing refinement, several macrocycles that included bulk solvent correction, anisotropic scaling of the data, individual coordinate refinement with minimization, and individual isotropic ADP (atomic displacement parameters) refinement were carried out with maximum likelihood as a target. In the course of the refinement, K-7174 compound and water molecules were added to the models by manual inspection of their positions in both 2*F*o-*F*c and *F*o-c maps, and individual ADP refinement was carried out in the final stages. Map fitting and other manipulations with molecular models were performed using the graphic software COOT [34]. The stereochemistry of the final models was assessed using MolProbity [35]. Data collection and refinement statistics are summarized in Table 2. Atomic coordinates and structure factors of the complex have been deposited in the Protein Data Bank under accession code 4EU2.

**Table 2:** Statistics of crystallographic analysis.

Space group/unit cell(Å)	*P*21/*a = *134.26, *b = *301.36, *c = *143.96, *ß* = 112.9°
Resolution range (Å)	50.0–2.5
Reflections (Measured/Unique)	313,772/962,020
Completeness (Overall/Outer Shell, %) a	88.5/72.3
*R-*merge (Overall/Outer Shell, %)a, b	10.1/28.0
Redundancy (Overall)	3.1
Mean <Ió/(I)> (Overall)	12.2
Overall *B*-factor from Wilson plot (Å^2^)	22
**Refinement statistics**	
*R-work* c/*R-free* (%)^c^	20.2/25.5
R.m.s.d. bond lengths/bond angles (Å)	0.008/1.178
Average *B*-factor (protein/water/compound, Å^2^)	39/31/82
Ramachandran plot	
Favored/allowed/outlier (%)	95.86/3.82/0.32

a Completeness and *R-*merge, are given for overall data and for the highest resolution shell.

The highest resolution shells for the dataset were 2.54–2.50 Å.

b *R*merge = ∑ | *I_i_* – <*I*> |/∑|*I_i_*|; where *I_i_* is intensity of an observation and <*I*> is the mean value of that reflection and the summations are over all equivalents.

c *R-work* = ∑*_h_* || *Fo(h)* | – | *Fc(h)* ||/∑*_h_Fo(h)*; where *Fo* and *Fc* are the observed and calculated structure factor amplitudes, respectively. The *R-free* was calculated with 5% of the data excluded from the refinement.

### Establishment of Bortezomib-resistant MM Cell Lines

To confer bortezomib resistance to MM cells, we transduced with mutated *PSMB5* cDNA as described previously [17]. A mutation was inserted at nucleotide position 322 (G/A) by PCR-based site directed mutagenesis using wild-type *PSMB5* cDNA (obtained from OpenBiosystems, Thermo Fisher Scientific, Huntsville, AL) as a template. Mutated *PSMB5* cDNA was inserted into a lentiviral vector CSII-CMV-MCS-IRES-VENUS (kindly provided by Dr. Hiroyuki Miyoshi, RIKEN BioResorce Center, Ibaraki, Japan) [36]. Wild-type *PSMB5* cDNA was also inserted into the same vector and used as a control. These vectors were co-transfected into 293FT cells with packaging plasmids (Invitrogen, Carlsbad, CA) to produce infective lentiviruses in culture supernatants as previously described [37]. RPMI8226 cells were infected with each viral supernatant for 24 h. We collected VENUS-positive cells using a FACSaria flow cytometer (Becton Dickinson, Bedford, MA) and seeded them at one cell/well in a 96-well plate to obtain single cell clones.

### Isobologram Analysis of Drug Interaction

The cytotoxic interaction of bortezomib and K-7174 was evaluated at the point of IC_80_ by the isobologram of Steel and Peckham. The IC_80_ was defined as the concentration of drugs that produced 80% inhibition of cell growth. The theoretical basis of the isobologram method has been described in detail previously [38]. Briefly, the envelope of additivity is constructed from dose–response curves of the combined drugs. The combination is regarded as additive when the data points of the drug combination fall within the envelope of additivity. The drug combination is regarded as supra-additive (synergism) and antagonistic when the data points fall to the left and the right of the envelope, respectively.

## Results

### Homopiperazine Derivatives Inhibit Proteasome Activities

**Figure 1:**
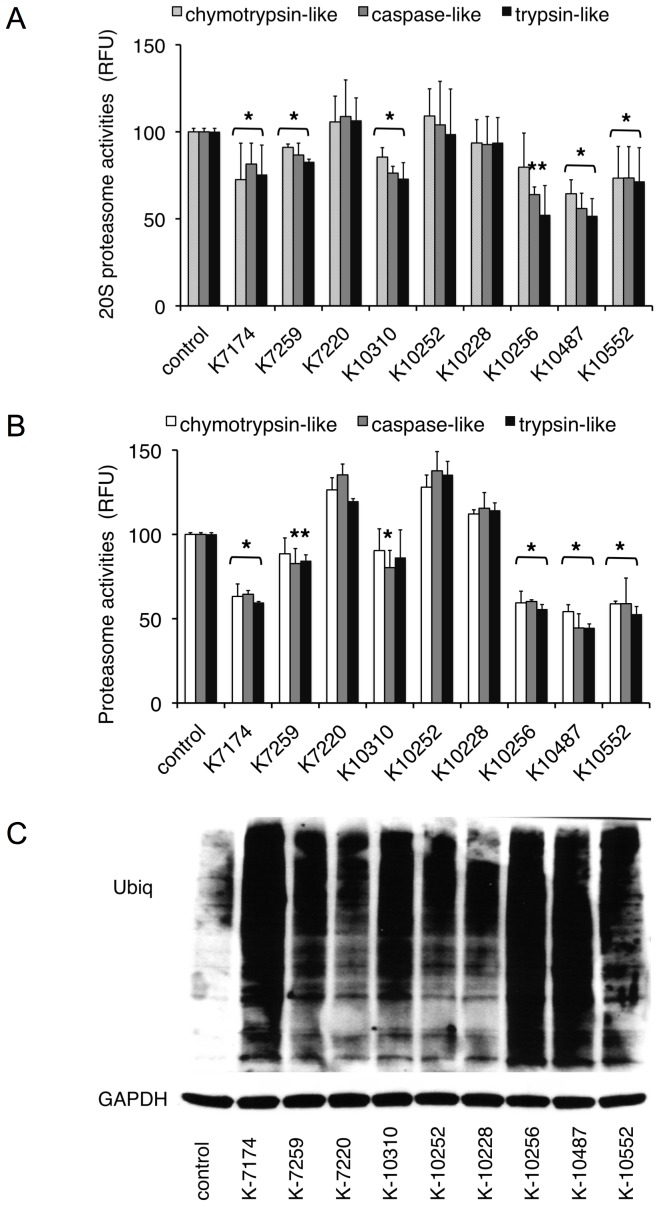
Inhibition of 20 S proteasome activity by homopiperazine derivatives. A. Purified erythrocyte-derived 20 S proteasome was incubated in the absence (control) or presence of the indicated HPDS at 5 µM. Chymotrypsin-like, caspase-like and trypsin-like activities were determined by measuring fluorescence generated from the cleavage of specific substrates. Results are represented as relative fluorescence units (RFU) with control set at 100%. The means ± S.D. (bars) of three independent experiments are shown. *P*-values were calculated by one-way ANOVA with the Student-Newman-Keuls multiple comparisons test. Asterisks indicate p<0.05 against corresponding controls. B. RPMI8226 cells were treated with or without 5 µM HPDs, and analyzed for proteolytic activities as described above. C. RPMI8226 cells were cultured in the absence (control) or presence of 10 µM HPDs for 24 hours, and subjected to immunoblotting for ubiquitinated proteins and GAPDH (internal control).

The ability of K-7174 to elicit the UPR prompted us to speculate that this drug could inhibit proteasome activity [5], [6], [24]. To test this idea, we incubated purified human erythrocyte-derived 20 S proteasome with K-7174 and eight HPDs, which are structurally similar to K-7174 (Table 1), and measured chymotrypsin-like (ß5), caspase-like (ß1), and trypsin-like (ß2) activities of the proteasome using specific fluorogenic peptides. As shown in Fig. 1A, six out of nine HPDs significantly inhibited proteasome activities of the purified 20 S proteasome. We performed the same experiments with cell-based assays using RPMI8226 cells to confirm the proteasome-inhibitory effect of the six HPDs (Fig. 1B). Interestingly, HPDs inhibited all three catalytic subunits with similar kinetics, suggesting a different mechanism of action from bortezomib.

Among the HPDs examined, K-7174, K-10256, K-10487 and K-10552 showed relatively strong effects on proteasome activities. They commonly possess methoxy-phenyl groups and pentenyl arms (with five carbon atom chains; C = 5) (Table 1). In contrast, proteasome-inhibitory activity was weak or none in K-7259 and K-7220, which have shorter arms (C = 4 and 3, respectively), and K-10310 and K-10252, which have longer arms (C = 6 and 7, respectively), although all of them possess trimethoxy-phenyl groups (Table 1). The inhibitory effect was also undetected in K-10228, which has trimethyl-phenyl groups instead of trimethoxy-phenyl groups and pentenyl arms (C = 5) (Fig. 1A and B). Consistent with these data, immunoblot analysis revealed a marked accumulation of ubiquitinated proteins in RPMI8226 cells treated with K-7174, K-10256, K-10487 and K-10552 but not K-7259, K-7220, K-10252 and K-10228 (Fig. 1C). These results suggest that the trimethoxy-phenyl group at the tip of the pentenyl arm (C = 5) is a critical structure of homopiperazine-derived proteasome inhibitors. Based on this finding, K-7174 was selected as the most promising candidate for pharmaceutical development as a PI and was further characterized in this study.

### K-7174 Inhibits Proteasome Activities in a Distinct Manner from Bortezomib

**Figure 2:**
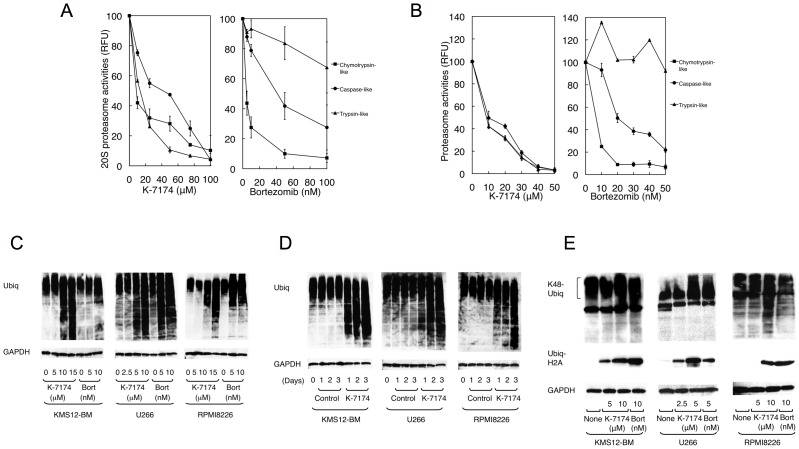
Inhibition of 20S proteasome activity by K-7174. A. We treated purified erythrocyte-derived 20 S proteasome with either K-7174 or bortezomib at the indicated doses and determined chymotrypsin-like, caspase-like and trypsin-like activities as described in the legend of Fig. 1. B. RPMI8226 cells were treated with either K-7174 or bortezomib at the indicated doses, and analyzed for proteolytic activities. C. MM cell lines (KMS12-BM, U266, and RPMI8226) were cultured with K-7174 or bortezomib (Bort) at the indicated doses for 48 hours. Whole cell lysates were subjected to immunoblotting for ubiquitinated proteins and GAPDH (internal control). D. MM cell lines were cultured with either K-7174 (5 µM for U266 and 10 µM for KMS12-BM and RPMI8226) or the vehicle alone (Control) for up to 3 days. Whole cell lysates were prepared at given time points and subjected to immunoblotting as described above. E. MM cell lines were cultured in the absence (None) or presence of K-7174 or bortezomib (Bort) at the indicated doses for 48 hours, and subjected to immunoblotting for lysine48-linked polyubiquitinated proteins (K48-Ubiq), ubiquityl histone H2A (Ubiq-H2A), and GAPDH (internal control).

First, we compared the proteasome-inhibitory activity of K-7174 with that of bortezomib. As shown in Fig. 2A, K-7174 similarly inhibited all three subunits in a dose-dependent fashion, whereas bortezomib did not affect trypsin-like activity but efficiently inhibited chymotrypsin- and caspase-like activities. This pattern was readily reproducible in cell-based assays using RPMI8226 (Fig. 2B) and other myeloma cell lines (data not shown).

Next, we monitored the accumulation of ubiquitinated proteins in K-7174-treated MM cells in comparison with bortezomib. As shown in Fig. 2C and D, K-7174 induced a marked accumulation of ubiquitinated proteins in all three cell lines in a dose- and time-dependent fashion, as did bortezomib. In addition, we detected the accumulation of lysine 48-linked polyubiquitinated proteins and ubiquityl histone H2A, both of which represent specific and critical modifications leading to proteasomal degradation, in both K-7174- and bortezomib-treated MM cells (Fig. 2E). These results indicate that K-7174 is a novel PI distinct from bortezomib in its chemical structure and effects on proteasome activity.

### K-7174 Interacts with the Catalytic Domains of ß1, ß2 and ß5 Subunits of the Proteasome in a Distinct Manner from Bortezomib

**Figure 3:**
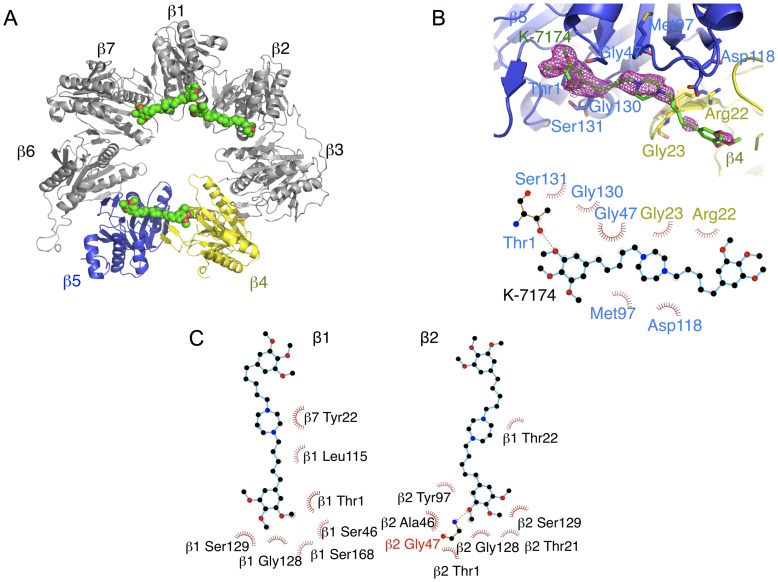
Crystallographic structure of the K-7174/proteasome complex. A. An overall ribbon diagram showing the folds of ß1 to ß7 subunits in the proteasome. The ß4 and ß5 subunits are colored yellow and blue, respectively. The K-7174 molecules bound to each ß subunit are shown as a space-filling representation colored green. Red spots indicate oxygen atoms. B. The final averaged electron density map (2*F*o-*F*c) covering K-7174 is shown (contoured at 1∂) (upper panel). A schematic diagram showing the interactions between K-7174 and the ß4 and ß5 subunits. The residues of ß4 and ß5 subunits associated with K-7174 are colored yellow and blue, respectively. A hydrogen bond is shown as a green dotted line (lower panel). C. A schematic diagram showing the interactions between K-7174 and the ß1 and ß2 subunits. The residues of ß1 and ß2 subunits associated with K-7174 are shown. A hydrogen bond is shown as a green dotted line.

To understand the mechanisms of proteasome inhibition by K-7174, we determined the X-ray crystal structure of the yeast 20 S proteasome in complex with K-7174 at 2.5 Å resolution (Table 2). Analysis of the structure revealed that three molecules of K-7174 bind to and block the active sites of all three catalytic ß-type subunits, ß1, ß2 and ß5, with a similar binding mode (Fig. 3A), consistent with the biochemical data. Figure 3B shows the conformation and binding mode of K-7174 near the ß5 active site for example. The electron density of K-7174 was well-defined except for one trimethoxy-phenyl group near the ß4 subunit, which was partially discorded (Fig. 3B). The overall binding is determined largely by hydrophobic interactions between K-7174 and Gly47, Met97, Asp118, Gly130 and Ser131 of the ß5 subunit as well as Arg22 and Gly23 of the ß4 subunit (Fig. 3B). Importantly, the oxygen atom of the methoxy group of K-7174 makes a hydrogen bond (with a distance of 2.9 Å) to the OH group of the N-terminal threonine residue (Thr1), which acts as a nucleophile in hydrolysis. We also observed that, despite some difference in binding interactions, the active sites of the ß1 and ß2 subunits are blocked by the trimethoxy-phenyl group of K-7174 in a similar fashion to that observed in the ß5 subunit (Fig. 3C). These findings are fully compatible with our conclusion from biochemical analyses, and confirmed that the trimethoxy-phenyl group, but not the trimethyl-phenyl group, interacts with the active sites of three catalytic subunits via hydrogen bonding and the pentenyl arm (C - 5) fits the hydrophobic grooves between ß subunits via hydrophobic interaction. It is highly likely that K-10256, K-10487 and K-10552 interact with ß-subunits in a similar manner. Taken together, these results provide the molecular basis of HPDs’ ability to inhibit all three catalytic subunits at the same time.

### Cytotoxic Effects of Homopiperazine Derivatives on Hematological Malignancies *in vitro*

**Figure 4:**
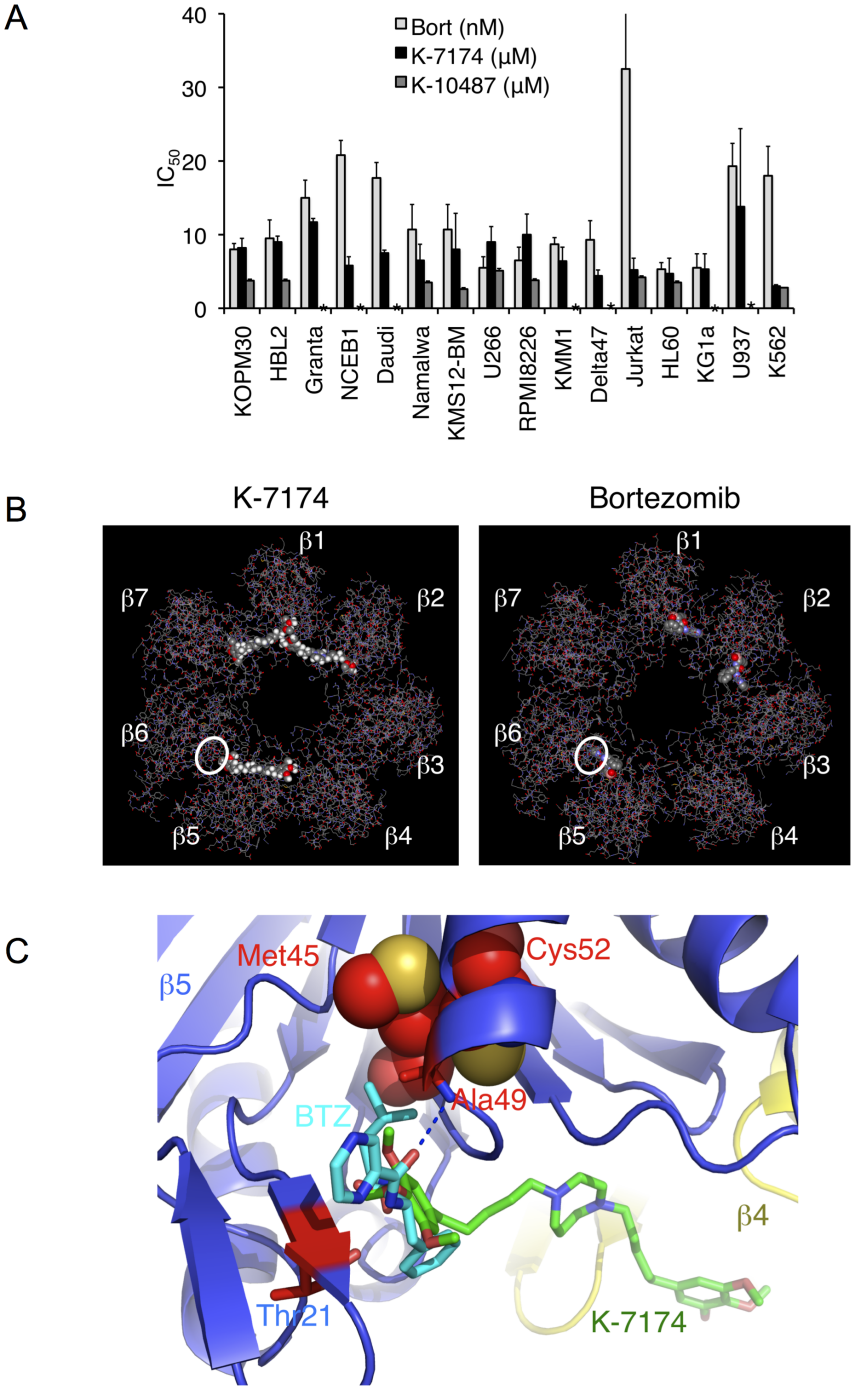
Comparison of K-7174 and bortezomib in cytotoxic activity and proteasome binding. A. Cell proliferation was measured by MTT assays after culturing with serially diluted K-7174, K-10487 and bortezomib for 72 hours. Absorbance at 450 nm was analyzed with a microplate reader, and expressed as a percentage of the value of corresponding untreated cells. The IC_50_ value was defined as the concentration of each drug that produces 50% inhibition of cell growth. The means ± S.D. (bars) of three independent experiments are shown. Asterisks indicate “not determined”. B. Overall crystallographic structures showing the folds of ß1 to ß7 subunits in the proteasome bound with K-7174 (left panel) and bortezomib (right panel). Mutation sites observed in bortezomib-resistant cells are circled. C. Structure of the proteasome in complex with bortezomib (PDB cord 2F16) overlapped that with K-7174 described here. Only the protein atoms of the bortezomib-bound form are shown. Bortezomib-resistant mutant residues (Ala49, Thr21, Cys52 and Met45) are colored red and shown as a space-filling diagram.

Given the proteasome-inhibitory action of HPDs, we investigated whether these agents exert cytotoxic activity against myeloma and other hematological malignancies. To this end, we cultured various cell lines with K-7174 and K-10487, and determined IC_50_ values using MTT assays. As shown in Fig. 4A, both agents showed remarkable cytotoxicity at ∼10 µM in most cell lines. The cytotoxic doses were virtually identical to the proteasome-inhibitory concentrations, suggesting that K-7174 and K-10487 exert cytotoxic effects mainly via proteasome inhibition. Consistent with this notion, the cytotoxic activity of both K-7174 and bortezomib was mediated via the caspase-8-dependent pathway (manuscript in preparation). However, the sensitivity pattern was obviously different between HPDs and bortezomib, implying a difference in their mechanisms of action.

### Comparison of the Binding Modes of K-7174 and Bortezomib

To gain mechanistic insights, we compared the binding mode of K-7174 with that of bortezomib using X-ray crystallographic data. As shown in Fig. 4B, three molecules of K-7174 bind to the active pockets of the ß1, ß2 and ß5 subunits along hydrophobic grooves in the direction of the ß7, ß1 and ß4 subunits, respectively. In contrast, bortezomib is attached to the ß5 subunit by a hydrogen-bond network composed of Ala49, Thr21 and Gly44 [39] (Fig. 4B and C). Mutations of amino acids within or near the bortezomib-binding pocket in the ß5 subunit, such as Ala49, Thr21, Met45 and Cys52, were reported to cause bortezomib resistance by reducing the affinity to the drug (14–19). Among them, Ala49 makes a direct hydrogen bond to bortezomib, explaining why this position is most frequently mutated in bortezomib-resistant cells. Furthermore, a Cys52Phe or Met45Val substitution results in a steric clash between the side chains of these two residues (Fig. 4C), leading to repulsion of bortezomib from the binding pocket [14], [16], [17]. In contrast, these mutations should not affect the affinity of K-7174 to ß5 subunit, because the binding site of K-7174 is spatially distinct from the bortezomib-binding pocket (Fig. 4B and C).

### K-7174 Overcomes Bortezomib Resistance caused by a ß5-subunit Mutation

**Figure 5:**
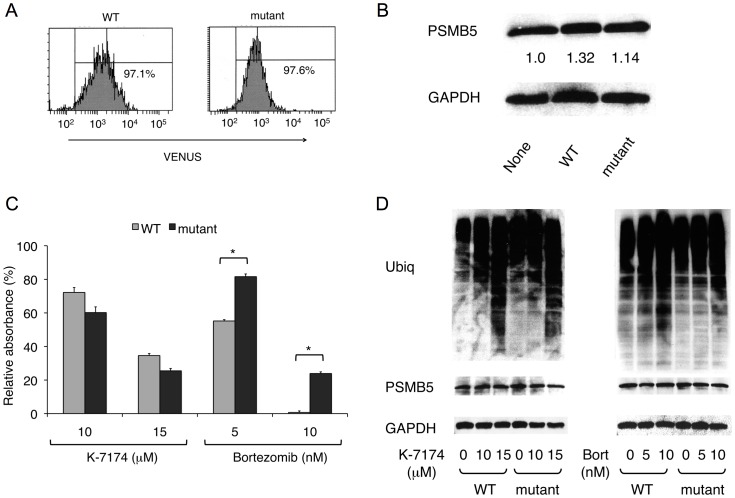
Cytotoxic effects of K-7174 on bortezomib-resistant MM cells. We established wild-type (WT) and mutant (mutant) sublines from RPMI8226 by transducing with wild-type and mutated *PSMB5* cDNA, respectively, and analyzed the expression of VENUS by flow cytometry (A) and proteasome ß5 subunit by immunoblotting (B). The signal intensities of ß5 subunit (PSMB5) were quantified, normalized to those of the corresponding GAPDH, and shown as relative values in the panel B. C. Cell proliferation was measured by MTT assays after culturing each subline with either K-7174 or bortezomib at the indicated doses for 72 hours. Results are represented as relative absorbance with untreated control set at 100%. The means ± S.D. (bars) of three independent experiments are shown. *P*-values were calculated by one-way ANOVA with the Student-Newman-Keuls multiple comparisons test. Asterisks indicate p<0.01 against the WT subline. D. Each subline was cultured with either K-7174 or bortezomib (Bort) at the indicated doses for 48 hours. Whole cell lysates were subjected to immunoblotting for cellular protein ubiquitination, proteasome ß5 subunit (PSMB5) and GAPDH (internal control).

Because K-7174 appears to inhibit proteasome activity with a distinct mode from bortezomib, it is anticipated that K-7174 is effective for bortezomib-resistant cells. Previous studies revealed that a mutation of the *PSMB5* gene at nucleotide position 322 (G322A), which corresponds to the substitution of Ala49 to Thr (Ala49Thr), induced conformational changes in the bortezomib-binding pocket of ß5 subunit and was responsible for acquired bortezomib resistance in T-cell acute lymphoblastic leukemia and myeloid leukemia cells [14]–[16]. Recently, Ri *et al*. [17] reported the establishment of bortezomib-resistant MM cell lines by transduction with G322A-mutated *PSMB5* cDNA. Taking the same approach, we established three mutant sublines (mutant-5B, 5F and 9B) from RPMI8226 cells lentivirally transduced with mutated *PSMB5* along with a marker gene *VENUS*. As controls, three wild-type sublines (WT-4G, 8F and 9G) were concomitantly established by mock transfection. We selected mutant-5F and WT-9G as representative sublines after determining the sensitivity to bortezomib (data not shown). The expression levels of VENUS and PSMB5 were virtually identical in these sublines (Fig. 5, A and B). As expected, sensitivity to bortezomib was significantly lower in the mutant subline than in the WT subline in MTT assays (Fig. 5C). In striking contrast, K-7174 induced cytotoxicity equally in WT and mutant sublines (Fig. 5C). In correlation with the results of MTT assays, K-7174 inhibited chymotrypsin-like (ß5) activity similarly in both sublines, whereas bortezomib could only partially inhibit the activity in the mutant subline (data not shown). These results were fully reproducible in other WT and mutant sublines (data not shown). To confirm the effects on proteasome activities, we determined the accumulation of ubiquitinated proteins in these sublines. As shown in Fig. 5D, ubiquitinated proteins were accumulated to a lesser extent in mutant cells than WT cells when they were treated with bortezomib (right panel). In contrast, K-7174 similarly induced intracellular protein ubiquitination in WT and mutant sublines (Fig. 5D, left panel). These results suggest that K-7174 can overcome bortezomib resistance.

### K-7174 and Bortezomib Exert Additive Cytotoxicity Against MM Cells

**Figure 6:**
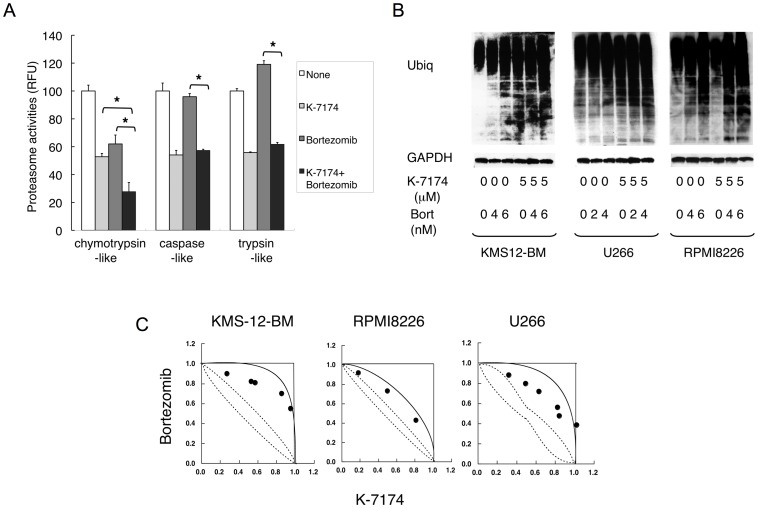
K-7174 and bortezomib exert additive cytotoxicity against MM cell. A. We treated RPMI8226 cells with K-7174 (10 µM), bortezomib (5 nM) or both agents, and determined chymotrypsin-like, caspase-like and trypsin-like activities. Results are represented as relative fluorescence units (RFU) with vehicle controls set at 100%. The means ± S.D. (bars) of three independent experiments are shown. Asterisks indicate p<0.01 by paired Student’s *t*-test. B. We cultured KMS12-BM, U266 and RPMI8226 cells in the absence or presence of K-7174, bortezomib or both agents for 48 hours at the indicated doses. Whole cell lysates were subjected to immunoblotting for ubiquitinated proteins and GAPDH (internal control). Data are representative of multiple independent experiments. C. Isobolograms of simultaneous exposure of three MM cell lines to K-7174 and bortezomib. The concentrations that produced 80% growth inhibition are expressed as 1.0 on the ordinate and abscissa of isobolograms. The envelope of additivity, surrounded by solid and broken lines, was constructed from dose–response curves of bortezomib and K-7174. The combination is regarded as additive, because all data points fall within the envelope of additivity. The isobolograms shown are representative of at least three independent experiments. Each point represents the mean of at least three independent experiments; standard deviations were less than 25% and were omitted.

As K-7174 inhibits proteasome activity in a distinct manner from bortezomib, their combination would be additive and useful for dose reduction of bortezomib. Indeed, K-7174 additively enhanced the proteasomal ß5-inhibitory effect of bortezomib (Fig. 6A) as well as bortezomib-induced accumulation of ubiquitinated proteins (Fig. 6B). Furthermore, isobologram analysis of drug interaction revealed that K-7174 and bortezomib showed additive cytotoxicity in MM cell lines (Fig. 6C).

## Discussion

In the present study, we show that HPDs constitute a novel class of PIs with a unique mode of proteasome binding. Although many kinds of small molecular PIs with various chemical structures have been developed [40], [41], this is the first demonstration of the proteasome-inhibitory activity of HPDs. In addition, most of the previous PIs mainly acted on one or two catalytic subunits and their mechanisms of action are not fully understood [40], [41]. In contrast, we have demonstrated that HPDs act on all three catalytic subunits of the proteasome by direct binding to the active pockets of the ß1, ß2 and ß5 subunits with a similar binding mode and kinetics. These results indicate the unique features of homopiperazine-derived PIs in chemical structures and effects on the proteasome. Moreover, we have identified the critical chemical structure of homopiperazine-derived PIs; therefore, these observations may contribute to the development of novel PIs with higher activity and specificity.

The high concentrations to trigger cytotoxicity might be the obstacle for clinical application of K-7174. Crystal structure analyses revealed that K-7174 interacts with ß subunits largely via hydrophobic interaction, whereas bortezomib binds to the ß5 subunit via a hydrogen-bond network, explaining why higher concentrations are required for HPDs compared with bortezomib. Therefore, the development of novel HPDs with higher activity and specificity is essential for clinical translation. Our finding on the chemical structure of homopiperazine-derived PIs may be of great help in this regard.

Despite the great success of bortezomib in the treatment of refractory malignancies such as MM and mantle cell lymphoma [3], [4], we still intend to develop orally bioavailable PIs with distinct mechanisms of action from bortezomib. Several novel PIs, such as carfilzomib [42], [43], NPI-0052 [44], CEP-18770 [45], MLN9708 [46], and ONX-0912 [47], are now undergoing clinical trials and show considerable benefits for refractory/relapsed cases as well as untreated MM patients. Among them, carfilzomib and its derivative ONX-0912 are peptide derivatives and have greater selectivity for the ß5 subunit than bortezomib. Although NPI-0052 is a non-peptide PI targeting all three proteasome subunits, its effect was strong for chymotrypsin-like (ß5), moderate for trypsin-like (ß2), and weak for caspase-like (ß1) activities [44]. In addition, NPI-0052 is intravenously administered in clinical studies [48], although it is expected to have oral bioactivity [44]. MLN9708 is orally available and its efficacy has been demonstrated in phase I clinical trials with oral administration [49]; however, this drug is speculated to be ineffective for MM carrying ß5-subunit mutations because of its boronate-based structure similar to bortezomib. Recently, in contrast to our speculation, Chauhan et al. [50] reported the effectiveness of MLN9708 to overcome bortezomib resistance. As several mechanisms have been proposed for bortezomib resistance in addition to ß5 subunit mutations [51], MLN9708 may be effective for such cases.

HPDs are expected to compensate for the weak points of bortezomib as well as the second generation PIs described above, because HPDs are non-peptide agents that inhibit all three catalytic subunits of the proteasome with equal kinetics and could be orally bioactive. Moreover, crystal structure analyses indicate that the binding mode is completely different from that of bortezomib [39] and NPI-0052 [52]. This ensures the activity of this agent against bortezomib-resistant cells, which was experimentally proven in this study, and probably against cells developing the resistance to NPI-0052. Moreover, we have found that oral administration of K-7174 is indeed effective and is not associated with obvious toxicities, including leukocytopenia, in a murine xenograft model (manuscript in preparation). These features provide a rationale for the clinical translation of HPDs as novel PIs with effectiveness for the treatment of bortezomib-resistant patients, a low probability of acquired drug resistance, and flexibility in dosing schedules.
